# Integration of lncRNA and mRNA Transcriptome Analyses Reveals Genes and Pathways Potentially Involved in Calf Intestinal Growth and Development during the Early Weeks of Life

**DOI:** 10.3390/genes9030142

**Published:** 2018-03-05

**Authors:** Eveline M. Ibeagha-Awemu, Duy N. Do, Pier-Luc Dudemaine, Bridget E. Fomenky, Nathalie Bissonnette

**Affiliations:** 1 Agriculture and Agri-Food Canada, Sherbrooke Research and Development Centre, Sherbrooke, QC J1M 0C8, Canada; duyngoc.do@agr.gc.ca (D.N.D.); pier-luc.dudemaine@agr.gc.ca (P.-L.D.); bridget.fomenky@agr.gc.ca (B.E.F.); nathalie.bissonnette@agr.gc.ca (N.B.); 2 Department of Animal Science, McGill University, Ste-Anne-De Bellevue, QC H9X 3V9, Canada; 3 Département des Sciences Animales, Université Laval, Québec, QC G1V 0A9, Canada

**Keywords:** calf, rumen, ileum, gastrointestinal tract, lncRNA, mRNA, pathways, *cis* target gene

## Abstract

A better understanding of the factors that regulate growth and immune response of the gastrointestinal tract (GIT) of calves will promote informed management practices in calf rearing. This study aimed to explore genomics (messenger RNA (mRNA)) and epigenomics (long non-coding RNA (lncRNA)) mechanisms regulating the development of the rumen and ileum in calves. Thirty-two calves (≈5-days-old) were reared for 96 days following standard procedures. Sixteen calves were humanely euthanized on experiment day 33 (D33) (pre-weaning) and another 16 on D96 (post-weaning) for collection of ileum and rumen tissues. RNA from tissues was subjected to next generation sequencing and 3310 and 4217 mRNAs were differentially expressed (DE) between D33 and D96 in ileum and rumen tissues, respectively. Gene ontology and pathways enrichment of DE genes confirmed their roles in developmental processes, immunity and lipid metabolism. A total of 1568 (63 known and 1505 novel) and 4243 (88 known and 4155 novel) lncRNAs were detected in ileum and rumen tissues, respectively. *Cis* target gene analysis identified *BMPR1A*, an important gene for a GIT disease (juvenile polyposis syndrome) in humans, as a candidate *cis* target gene for lncRNAs in both tissues. LncRNA *cis* target gene enrichment suggested that lncRNAs might regulate growth and development in both tissues as well as posttranscriptional gene silencing by RNA or microRNA processing in rumen, or disease resistance mechanisms in ileum. This study provides a catalog of bovine lncRNAs and set a baseline for exploring their functions in calf GIT development.

## 1. Introduction

From birth, calves undergo major physiological and dietary changes including adaptation to extrauterine life (first week of life), pre-ruminant stage (3 to 5 months of age) and weaning [1]. After birth, sufficient colostrum ingestion and microbial colonization is crucial for adequate development of the gastro-intestinal tract (GIT) and mucosal immune system [1,2]. These developmental transitions are accompanied by rapid changes in gene expression controlled by signal-mediated coordination of transcriptional and post-transcriptional mechanisms [3]. The transcriptional mechanisms include the activities of non-coding RNA (ncRNA) and transcription factors as well as epigenetic modifications. While only about 2% of the mammalian genome is transcribed as proteins, about 75–90% is transcribed as ncRNA, the vast majority being long non-coding RNA (lncRNA) [4].

LncRNAs are largely defined as RNA transcripts greater than 200 bp and possess no apparent protein coding potential [4,5]. LncRNAs are involved in the regulation of gene expression through diverse mechanisms including epigenetic modification of DNA, alternative splicing, posttranscriptional gene regulation, messenger RNA (mRNA) stability and translation [6,7,8]. Aided by deep and more sensitive sequencing technologies and computational prediction techniques, the catalogue of lncRNAs of mammalian species continues to rise. In humans, the number of lncRNA genes outstrips protein coding genes [9]. Increasing research has shown roles for lncRNA in many biological processes including genome imprinting, genome regulation, cellular differentiation and development, cardiac development and aging, regulation of innate immune response and disease [10,11,12,13]. LncRNAs can function in *cis* (*cis*-regulation), at the site of transcription, or in *trans* (*trans*-regulation) and as such participates in multiple networks regulating gene expression and function. The actions of lncRNA controls cell fate during development and dysregulation of these processes are known to underlie some disorders in humans as a result of chromosomal deletions and translocations [14].

A number of studies have provided evidence that lncRNAs are crucial players in cell differentiation and development including developmental regulation of pluripotency, differentiation, specialization, homeostasis, metamorphosis and embryonic development [11,14,15,16,17,18]. Analysis of lncRNA knockout mouse models reveals peri- and postnatal lethal phenotypes, incomplete penetrance, growth defects and defects in the lung, gastrointestinal tract and heart of neonates [19]. In particular, altered expression of several lncRNAs including F19, HOTAIR, ANRIL, FENDRR, etc., are linked with gastric and colon cancer [13]. In addition to tissue specificity of lncRNA expression [4,20,21], it is evident that they play roles in the development of the GIT [22,23,24].

Out of the thousands of lncRNAs identified in humans and other species, distinct roles have been characterized for just a small number [25,26,27] while roles for the vast majority are unknown. In bovine and other farm animal species, only a few studies have attempted a characterization of lncRNA transcripts. A pioneer study characterized the bovine ncRNA transcriptome and identified 23,060 bovine ncRNAs which were primarily intergenic, conserved and had modest correlation with protein coding genes [28]. Analyses of deep RNA sequence data identified 4848 and 584 lncRNAs, mainly intergenic lncRNAs in bovine skin and muscle, respectively [29,30,31]. A large number of lncRNAs (4227) including 18 highly expressed and 26 differentially regulated by diet supplemented with 5% linseed oil were identified in bovine mammary gland [32]. Another study identified 184 intergenic lncRNAs including 36 lncRNAs collocated with 172 milk related quantitative trait loci [33]. These data suggest regulatory roles of lncRNA in bovine.

In order to understand the possible roles of lncRNA during early development, we characterized and performed co-expression analyses of lncRNA and mRNA in the GIT of calves before and after weaning with a view to gaining insights into the occurrence, expression pattern and possible functions of lncRNAs during early growth. Clear understanding of the factors that regulate growth and immune maturation of the GIT of calves will promote informed calf management practices. The objectives of this study align with the mandate of the international consortium for Functional Annotation of Animal Genomes (FAANG, www.faang.org) [34,35], which is to detect functional regulatory elements in animal genomes necessary to understand how the genome is read and translated into complex phenotypes and thus fill the genotype-to-phenotype gap that is missing in current livestock improvement programs.

## 2. Materials and Methods 

### 2.1. Animals and Management

Procedures for animal management were according to the national codes of practice for the care and use of farm animals in research, teaching and testing [36] and approved by the animal care and ethics committee of Agriculture and Agri-Food Canada.

Thirty-two Holstein calves ≈5 days old were used. Calves were housed in individual pens and raised following standard management procedures for 96 days (experiment day 1 (D1) to D96). Animals were fed milk replacer at 6 L/day for the first four days and 9 L/day thereafter (Goliath XLR 27-16, La Coop, Montreal, QC, Canada) until weaning. Starter feed (Calf starter, Shur-Gain, St-Hyacinthe, QC, Canada) was introduced on D8. Calves were fed hay and starter feed after weaning. Hay, starter feed and water were provided ad libitum. Animals were weaned on D53 and it was gradually put in place; beginning from D43, milk replacer was reduced by half each day and weaning was complete (D53) when animals consumed at least 1 kg of starter feed for three consecutive days. Growth of animals was monitored by bi-weekly body measurements. On experiment D33 (pre-weaning), 16 calves were humanely euthanized and another 16 on D96 (post-weaning) for collection of ileum and rumen tissues. The tissues (mid-rumen and mid-ileum) were aseptically collected, rinsed in phosphate buffered saline to remove digesta, cut into small pieces, snap frozen in liquid nitrogen, transferred into 2 mL cryopreservation tubes and stored at −80°C until used. It should be noted that, eight ileum tissue samples of D33 were contaminated during transportation from the abattoir to the laboratory and were not further considered in this study; and for this reason, only eight ileum tissue samples of D96 were included in the analysis.

### 2.2. RNA Isolation

Total RNA from rumen (*n* = 32) and ileum (*n* = 16) tissues (30 mg/sample) was extracted using miRNeasy Kit (Qiagen Inc., Toronto, ON, Canada) following manufacturer’s recommendations. Briefly, tissues were homogenized in 700 µL TRIzol Reagent (Life Technologies, Burlington, ON, Canada) with a Polytron homogenizer (Polytron PT 10-35 GT, Kinematica AG, Luzern, Switzerland) using a 7 mm probe for 10 s at 12,000 rpm, repeated three times with incubation on ice between repetitions. Following 5 min incubation at room temperature, 140 µL chloroform was added to the mixture and vortexed vigorously for 20 s. The mix was centrifuged (15 min at 12,000× *g* at 4 °C) and 1.5 volumes ethanol (100%) was added to the aqueous phase. Samples were then transferred to the column and washed steps performed according to kit’s recommendations. Elution was done twice using 30 µL nuclease-free water each time. Total RNA (10 µg) was subjected to DNase treatment with Turbo DNA-free Kit (Ambion Inc. Foster City, CA, USA) to remove contaminating genomic DNA. Nanodrop ND-1000 (NanoDrop Technologies, Wilmington, DE, USA) was used to determine the concentration of RNA (before and after DNase treatment) and the integrity was assessed with Agilent 2100 Bioanalyzer (Agilent Technologies, Santa Clara, CA, USA) using the RNA 6000 Nano Labchip Kit (Agilent Technologies) after DNase treatment. All samples had a RNA integrity number (RIN) ≥8.0.

### 2.3. Library Preparation and RNA-Sequencing

Total RNA (2 µg) was subjected to ribosomal RNA (rRNA) depletion using Ribo-Zero Gold rRNA Removal Kit (Illumina Inc., San Diego, CA, USA) following company recommendations. Generation of libraries for sequencing was made using NEBNext Ultra Directional RNA Library Prep Kit for Illumina (New England Biolabs, Whitby, ON, Canada) with NEBNext Multiplex Oligos for Illumina^®^ (New England Biolabs) for barcoding the multiplexed samples. Library concentration was measured using the Quant-iT PicoGreen double-stranded DNA (dsDNA) Assay Kit (Life Technologies). Fragment size was estimated using High Sensitivity DNA Analysis Kit (Agilent) with Agilent 2100 Bioanalyzer (Agilent) and quantified by real time quantitative polymerase chain reaction (qPCR) using the Kapa Library Quantification Illumina/ABI Prism Kit protocol (KAPA Biosystems, Wilmington, MA, USA). Six libraries were pooled in equimolar amounts and paired-end sequenced (2 × 126 bp) in one lane on a High Throughput Model flow cell on an Illumina HiSeq 2500 system by The Centre for Applied Genomics, The Hospital for Sick Children, Toronto (http://www.tcag.ca/).

### 2.4. Sequence Data Processing, Alignment and Identification of Genes

Demultiplexed sequence files in fastq format were processed using a pipeline developed by McGill University and Genome Quebec Innovation Centre (MUGQIC, http://gqinnovationcenter.com/). Briefly, adaptor sequences were removed with Trimmomatic software v0.32 [37] which was set to keep reads longer than 32 bp with a minimum phred score of 30. Alignment of reads to the bovine genome (UMD3.1, v84) was accomplished with STAR v2.5.1b [38]. Following alignment, Picard tools [39] and RNA-SeQC [40] were used to generate quality control read metrics such as percentage of mapped reads and genomic localization (intronic, intergenic, etc.). Uniquely mapped and properly paired reads were used in transcript construction with Cufflinks (v2.2.1) [41] for mRNA identification. Constructed transcripts were compared with Ensembl bovine gene annotation (release 84) to identify expressed mRNAs using Cuffcompare [41].

For lncRNA identification, alignment files or properly mapped reads from all the samples of the same tissue were sorted and merged using Samtools v1.3.1 [42] prior to the transcript construction step with Cufflinks [41] to generate a unique set of all transcripts per tissue. To identify expressed lncRNAs, only transcripts >200 bp were kept and compared with Ensembl bovine gene annotation (release 84) to remove annotated transcripts (mRNA) and transcripts overlapping with other noncoding RNA species (transfer RNA (tRNA), rRNA, small nuclear RNA (snRNA), small nucleolar (snoRNA) and microRNA (miRNA)) using Cuffcompare [41]. Specifically, transcripts with class code “i” (predicted transcript fall entirely within a reference intron), “u” (predicted transcript is intergenic in comparison with known reference transcripts) and “x” (exon of predicted transcript overlaps reference but lies on the opposite strand) when compared against Ensembl-ncRNA databases were retained. Retained transcripts were then assessed for their coding potential using Coding-Non-Coding Index (CNCI) [43] and Coding-Potential Assessment Tool (CPAT) [44] and intersecting transcripts across CNCI score < 0 and CPAT score < 0.5 were blasted against the Swiss-prot database to further filter transcripts with the ability to code for a protein (evalue < 1 × 10^−5^) using usearch [45] and also transcripts that possessed an open reading frame with the ability to code for a peptide of 100 or more amino acids.

The retained transcripts were compared with known bovine lncRNA annotation from NONCODE2016 database [46] using Cuffcompare. Transcripts with class codes “=” (predicted transcript has exactly the same introns as the reference transcript), “c” (predicted transcript is contained within the reference transcript) and “j” (predicted transcript is a potential novel isoform that shares at least one splice junction with a reference transcript) were classified as known bovine lncRNAs while the rest were classified as novel lncRNAs. The expression of lncRNAs (known and novel) and mRNAs were quantified in each sample using HTSeq-count (v0.6.1p1) [47] with default settings (-s reverse).

### 2.5. Differential mRNA and lncRNA Expression Analyses

Differential gene expression (DE) was accomplished with DeSeq2 (v1.14.1) [48], an R package [49] that implements a negative binomial model in DE analysis. Those genes (mRNA or lncRNA) with DESeq2 normalized counts >5 and in at least 10% of libraries were considered truly expressed and were used in DE and pathway analyses. The differences in gene expression (mRNA or lncRNA) between D33 and D96 were considered significant at Benjamini and Hochberg [50] corrected *p*-value < 0.05.

### 2.6. Gene Ontology and Pathways Enrichment of Differentially Expressed mRNAs

Differentially expressed genes were enriched for gene ontology (GO) and Kyoto Enciclopedia of Genes and Genomes (KEGG) pathways using ClueGO [51]. In this enrichment analysis, a hypergeometric test was used. For GO enrichment, the Bovine GO database and Benjamini and Hochberg (BH) [50] correction for multiple testing controlled *p*-values and GO with corrected *p* < 0.05 was considered significantly enriched. Meanwhile, since there are no KEGG databases for bovine species in ClueGO, the human KEGG database was used as background and pathways with uncorrected *p* < 0.05 were considered significantly enriched.

### 2.7. Identification of lncRNA cis Target Genes and cis Target Gene Enrichment

Since lncRNAs can *cis* regulate mRNAs, we performed co-expression analysis between mRNAs and lncRNAs in 50 Kb flanking regions of identified lncRNAs. Firstly, we computed Pearson’s correlation coefficients between each pair of lncRNA–mRNA. The mRNAs having significant correlations with lncRNAs at p.BH < 0.05 were considered potential *cis* target genes for those lncRNAs. Potential *cis* target genes of lncRNAs were subjected to enrichment analysis with ClueGO using the same procedure as for DE mRNAs. The interaction between DE lncRNAs and their *cis* target genes were visualized using Cystoscope v3.0 [52].

### 2.8. Real Time Quantitative Polymerase Chain Reaction 

Real time quantitative PCR was performed to verify the expression levels of genes DE by the method of RNA-Seq. Four potential housekeeping genes (*PPIB*, *ATP5B*, *GPI* and *PGK1*) were randomly selected from the list of non-DE genes by RNA-Seq in both tissues. Two DE genes (*IFI6* and *OAS1X*) were randomly selected from the list of DE genes common to both tissues while three DE genes in ileum tissue (*OAS2*, *Mx1* and *UBA7*) and three DE genes in rumen tissue (*ACTA2*, *CA3* and *HERC6*) were randomly selected from the list of DE genes unique to these tissues. Moreover, two lncRNAs were selected from rumen DE lncRNAs (rXLOC_042149 and rXLOC_022071; rXLOC denotes lncRNAs expressed in the rumen). Transcript-specific primers for the two lncRNAs and 12 mRNAs were designed using Integrated DNA Technologies Assay tool for real time quantitative PCR (Integrated DNA Technologies Inc., Skokie, IL, USA) (Supplementary Table S1). Reverse transcription was performed with the SuperScript III Reverse Transcriptase (Life Technologies Inc., Burlington, ON, Canada), using aliquots (1 μg) of the same total RNA used in RNA-Seq. The complementary DNA (cDNA) samples were diluted to 20 ng/μL. Real-time PCR reaction mix was composed of 5 µL Power SYBR Green PCR Master Mix (Life Technologies Inc.), 3 µL cDNA and 0.1 U AmpErase Uracil *N*-Glycosylase (UNG) (Life Technologies). Forward and reverse primer concentrations in the mix are presented in Supplementary Table S1. StepOne Plus Real-Time PCR System (Life Technologies) was used to perform qPCR reactions. The thermal cycling conditions were composed of a step for UNG treatment at 25°C for 5 min followed by an initial denaturation/activation step at 95 °C for 10 min, 45 cycles at 95 °C for 30 s, 60 °C for 60 s. The experiments were carried out in triplicate for each data point. The relative quantification of gene expression was determined using the 2−ΔΔCt method [53]. Normalization with GPI and ATP5B as housekeeping genes was done prior to statistical analysis for ileum and rumen samples, respectively. The housekeeping genes (*GPI* and *ATP5B*) were tested and found to be the most stable out of the four potentially stably expressed genes in their respective tissues using NormFinder. Statistical analysis (Student’s *t*-test with homogeneous variances) was done using MIXED procedure and SAS software (Statistical Analysis System, release 9.4, 2002–2012, SAS Institute Inc., Cary, NC, USA).

## 3. Results

### 3.1. RNA-Sequencing and Identification of Expressed mRNA and lncRNA Genes in the Gastrointestinal Tract of Calves

Next-generation RNA sequencing of 48 libraries from ileum and rumen tissues of calves generated a total of 2.5 billion reads (Supplementary Table S2). Following adaptor trimming and size selection, 2.4 billion reads (96%) with length >32 bp and having a phred score >30 were further processed. Out of this number, 91.84% were successfully mapped to the bovine genome (UMD.3.1). Out of successfully mapped reads, 86.17% uniquely mapped reads (Supplementary Table S2) were used in transcript construction.

A total of 24,616 expressed mRNAs were retained after removing transcripts that were not annotated to Ensemble bovine gene annotation release 84. After further filtering of genes with <5 normalized read counts and present in less than 10% of libraries, 15,905 and 15,628 genes in the ileum and rumen tissues, respectively were retained for further analyses (Supplementary Table S3).

After filtering and coding potential evaluation steps, lncRNA transcripts with ≥5 normalized counts and present in ≥10% of libraries resulting in 1568 (63 known and 1505 novel) and 4243 (88 known and 4155 novel) lncRNAs were considered expressed in ileum and rumen tissues, respectively (Supplementary Table S4a–d) and used in further analyses. Among them, 51 lncRNAs were common to both tissues (Supplementary Table S4e).

### 3.2. Genomic Features and Characteristics of Expressed lncRNAs

Majority (44.70%) of identified lncRNA transcripts were 200–999 bp long followed by transcripts 1000–2499 bp long (29.25%) while transcripts >9999 bp constituted only 1.25% of identified lncRNAs (Supplementary Table S5a). Two-hundred or more lncRNA genes/transcripts were located on 22 chromosomes (Bta 1, 2, 3, 4, 5, 7, 8, 9, 11, 12, 13, 14, 15, 16, 18, 19, 21, 24, 26, 28, 29 and x) (Supplementary Table S5b). Identified lncRNA genes were composed of one to five transcripts with majority composed of one transcript (95.85%) (Supplementary Table S5c). LncRNA transcripts were composed of one to 15 exons (Supplementary Table S5c). LncRNAs were classified based on their genomic locations into 11 classes with the reference of Ensembl bovine protein coding gene annotation (release 84) and majority (93%) were intergenic lncRNAs located at >1 Kb away from the nearest mRNA (Supplementary Table S5d).

### 3.3. Differentially Expressed mRNAs and Enrichment Analyses 

**Figure 1:**
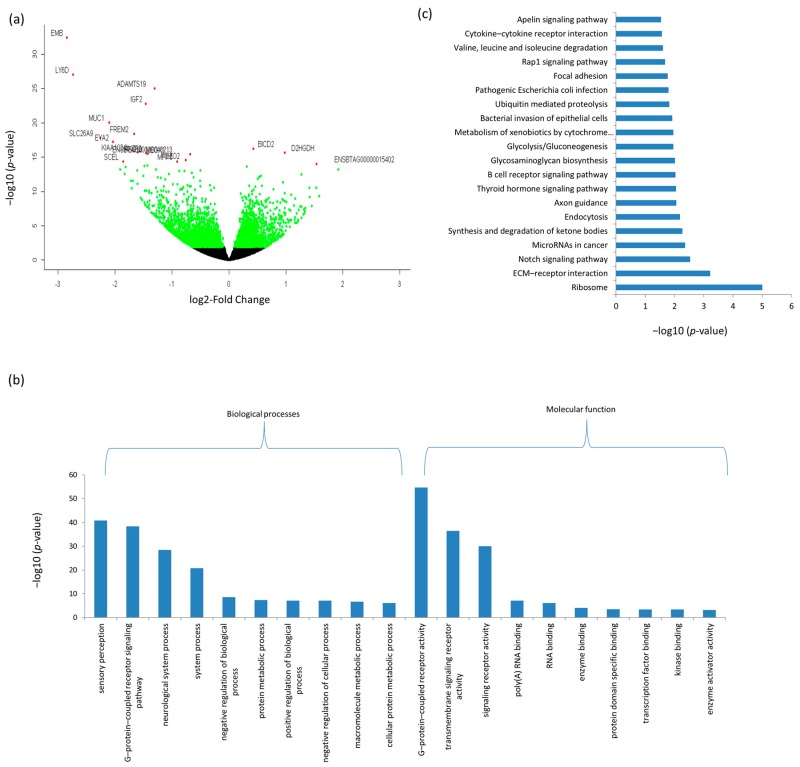
Differentially expressed genes and their enriched gene ontology (GO) terms and Kyoto Encyclopedia of Genes and Genomes (KEGG) pathways in rumen tissues. (**a**) Differentially expressed messenger RNAs (mRNAs); (**b**) top GO terms and (**c**) KEGG pathways. ECM: Extracellular matrix.

**Figure 2:**
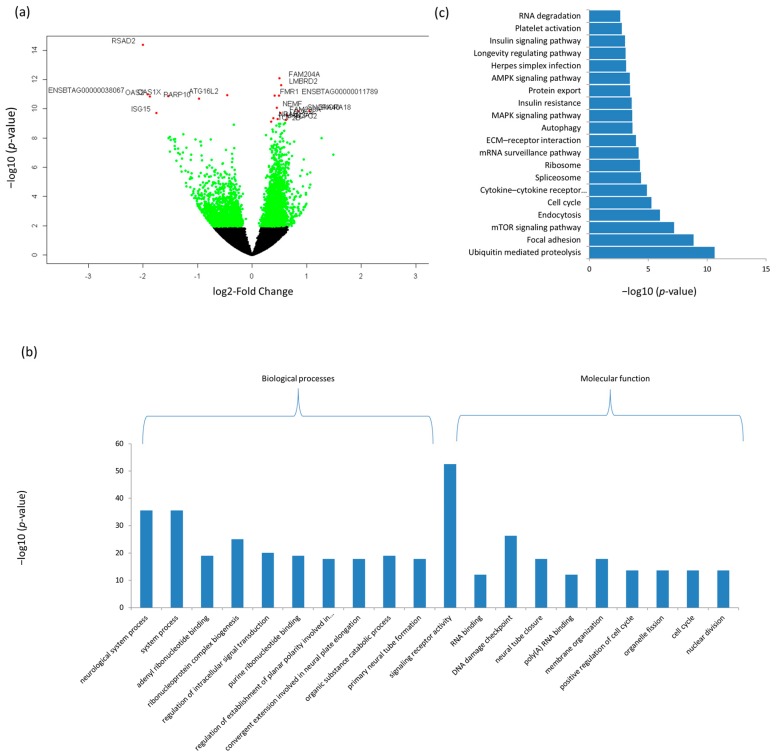
Differentially expressed genes and their enriched GO terms and KEGG pathways in ileum tissues. (**a**) Differentially expressed mRNAs; (**b**) top GO terms and (**c**) KEGG pathways. MAPK: Mitogen-activated protein kinase; mTOR: Mechanistic target of rapamycin.

To identify differentially expressed genes, raw read counts of retained transcripts (mRNA or lncRNA) were imported into DESeq2 [48]. DESeq2 normalizes read counts by calculating a size factor for each sample to correct for library size and RNA composition bias. A total of 3310 and 4217 mRNAs were DE between D33 and D96 in Ileum and rumen tissues, respectively (Figure 1a and Figure 2a, Supplementary Table S6a,b). Out of this number, 850 DE mRNAs were common to both tissues (Supplementary Table S6c). The top 20 most significant DE mRNAs are shown in Table 1 and Figure 1a and Figure 2a. Embigin (*EMB*) and radical *S*-adenosyl methionine domain containing 2 (*RSAD2*) were the most significant DE mRNAs for rumen and ileum tissues, respectively (Table 1).

A total of 459 biological process (BP) and 52 molecular function (MF) GO terms were significantly enriched for rumen DE mRNAs and the most enriched terms were sensory perception (*p* = 1.3 × 10^−41^) and G-protein coupled receptor activity (*p* = 2.4 × 10^−55^), respectively (Figure 1b and Supplementary Table S7a). Moreover, rumen DE mRNAs were also significantly enriched for 36 KEGG pathways and the most significantly enriched pathway was Ribosome (*p* = 1.00 × 10^−05^) (Figure 1c and Figure S1 and Table S7b).

A total of 590 BP-GO and 110 MF-GO terms were significantly enriched for ileum DE mRNAs and the most enriched terms were neurological system process (*p* = 2.2 × 10^−43^) and signaling receptor activity (*p* = 5 × 10^−50^), respectively (Figure 2b and Table S7c). Moreover, ileum DE mRNAs were significantly enriched for 86 KEGG pathways and the most significantly enriched pathway was Ubiquitin mediated proteolysis (*p* = 23.0 × 10^−12^) (Figure 2c and Table S7d).

### 3.4. Identification and Enrichment Analyses of *cis* Target Genes of lncRNAs

**Figure 3:**
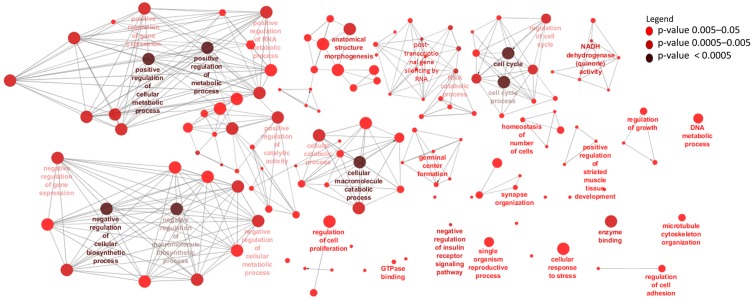
Gene ontology terms enriched for rumen long non-coding RNA (lncRNA) *cis* target genes. Only the relevant or highly significantly enriched terms are shown. See Supplementary Table S9a for detailed results. GTPase: Single GTPase (guanosine triphosphate); NADH: Nicotinamide adenine dinucleotide reduced form.

**Figure 4:**
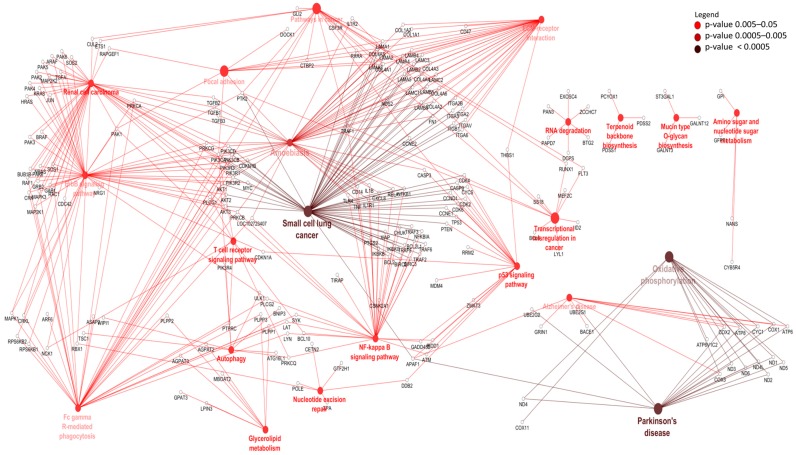
Kyoto Enciclopedia of Genes and Genomes pathways enriched for rumen lncRNA potential *cis* target genes. ErbB: Epidermal growth factor receptor; Fc: Fragment crystallisable region; NF-κB: Nuclear factor-κB.

To identify potential *cis* target genes of lncRNAs, a matrix of Pearson’s correlation was computed using the expression data of 15,628 mRNAs and 4243 lncRNAs and between 15,905 mRNA and 1567 lncRNAs for rumen and ileum tissues, respectively. A total of 632 mRNAs (within 50 Kb surrounding regions of lncRNAs) were significantly correlated (*p* < 0.05) with 884 lncRNAs and were considered their potential *cis* targets in rumen tissues (Supplementary Table S8a). Eight genes (*WWOX*, *TTLL7*, *STAG2*, *BMPR1A*, *NEDD4L*, *KIAAO319L*, *COA1* and *SUDS3*) were the potential *cis* targets of 27, 21, 20, 19, 18, 17, 16, 16 and 15 lncRNAs in rumen tissue, respectively while rXLOC_044298 and rXLOC_036836 potentially *cis* targeted 13 and 6 mRNAs, respectively (Supplementary Table S8a). GO enrichment analysis indicated that the 632 *cis* target genes of rumen lncRNAs were significantly enriched for 129 BP-GO and 13 MF-GO terms (Supplementary Table S9a, Figure 3) and 22 KEGG pathways (Supplementary Table S9b and Figure 4).

**Figure 5:**
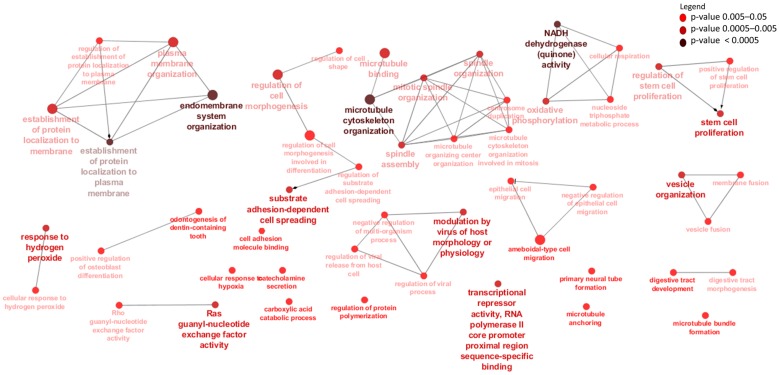
Gene ontology terms enriched for ileum lncRNA potential *cis* target genes.

**Figure 6:**
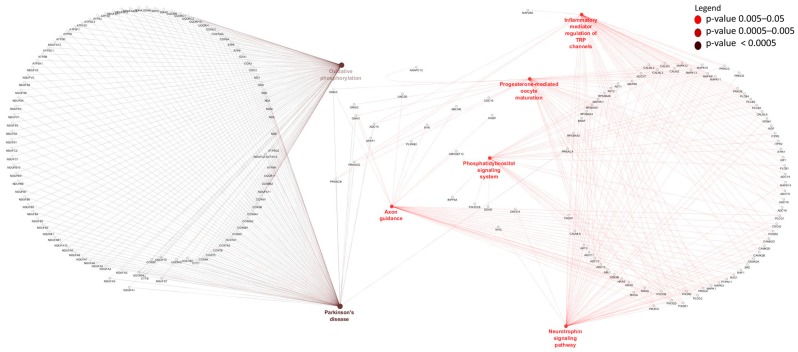
Kyoto Enciclopedia of Genes and Genomes pathways enriched for ileum lncRNA potential *cis* target genes. TRP: Transient receptor potential.

For ileum tissue, 610 lncRNAs potentially *cis* regulated 439 mRNAs (Supplementary Table S8b) and iXLOC_044298 and iXLOC_036836 (iXLOC denotes lncRNA identified in the ileum) potentially *cis* targeted 13 and 6 mRNAs while *BMPR1A* and *ST3GAL1* were potentially *cis* targeted by 21 and 18 different lncRNAs, respectively (Supplementary Table S8b). A total of 44 BP-GO and 7 MF-GO terms were enriched for the 407 potential *cis* genes of ileum lncRNAs (Supplementary Table S9c, Figure 5) while 8 KEGG pathways were significantly enriched (Supplementary Table S9d, Figure 6).

### 3.5. Differentially Expressed lncRNAs in Ileum and Rumen Tissues

**Figure 7:**
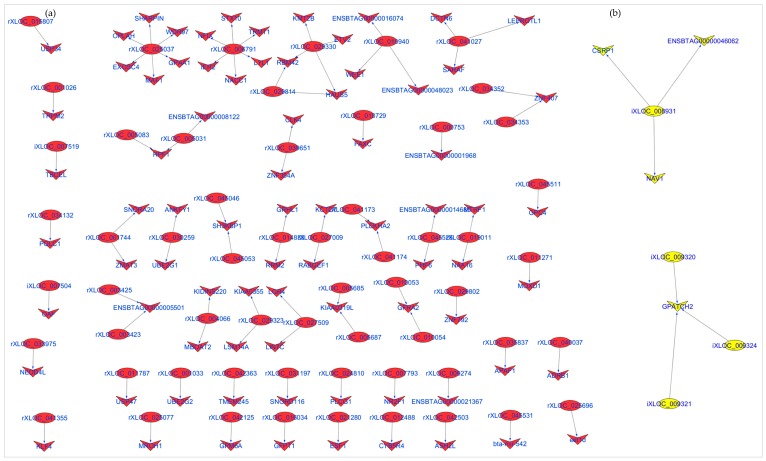
LncRNAs and their potential *cis* target genes in (**a**) rumen and (**b**) ileum tissues.

A total of 14 and 525 lncRNAs were significantly DE between D33 and D96 in ileum and rumen tissues, respectively (Table S10a,b). The top significant DE lncRNAs are shown in Table 2. rXLOC_027852 and iXLOC_002882 were the most significant DE lncRNAs in rumen and ileum tissues, respectively. For rumen, 57 out of 525 DE lncRNAs had potential *cis* roles by correlating significantly with 77 mRNAs (Supplementary Table S10c). Most of the DE lncRNAs had only one potential *cis* target mRNA while rXLOC_025037 and rXLOC_006791 had the most potential *cis* target mRNAs (Figure 7). For ileum, 7 out of 14 DE lncRNAs had potential *cis* regulatory roles, targeting (significant correlation) five different mRNAs (Table S10d) and iXLOC_008931 potentially *cis* targeted three different mRNAs (Figure 7). Furthermore, one mRNA was the potential *cis* target of three lncRNAs (iXLOC_009320, iXLOC_009324 and iXLOC009321) in ileum tissue (Figure 7). Some of the potential *cis* target genes of DE lncRNAs were also significantly DE between D33 and D96 (Table 3 and Figure 7).

### 3.6. Real Time Quantitative PCR Confirmation of RNA-Seq Results

**Figure 8:**
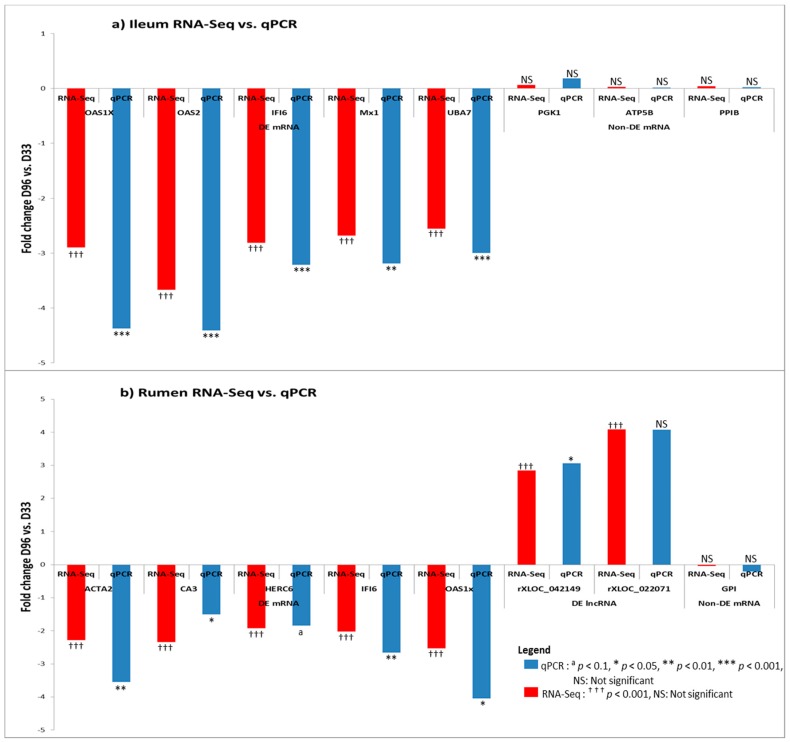
Quantitative real time PCR (qPCR) validation of the RNA-Sequencing (RNA-Seq) expression results of (**a**) mRNAs (five differentially expressed (DE) and three non-DE or potential housekeeping mRNAs) in ileum tissue and (**b**) mRNAs (five differentially expressed and two non-DE) and two lncRNAs in rumen tissue.

RNA-Seq results were mostly confirmed by qPCR analysis. For ileum samples, observed trend for all nine genes (four housekeeping and five DE genes) was similar to RNA-Seq results (Figure 8a). For rumen samples, the qPCR result was similar to RNA-Seq for four DE mRNAs, two lncRNAs and two housekeeping genes. However, one mRNA only showed a tendency towards significance (HERC6: *p* < 0.1) by the method of qPCR while one lncRNA (rXLOC_022071) was significantly DE by RNA-Seq but not by qPCR, although the fold change by both methods was the same (4.1-fold change, Supplementary Table S8b). Furthermore, two potential housekeeping genes PGK1 (*p* = 0.08) and PPIB (*p* = 0.06) in rumen tissue tended to be DE by the method of qPCR.

## 4. Discussion

### 4.1. Transcriptome mRNA Transition from Pre- to Post-Weaning Period in Rumen and Ileum Tissues

The rumen and ileum are important organs for the digestion and absorption of nutrients in ruminants. The molecular mechanisms and key drivers underlying developmental changes in rumen and ileum in the early part of life have been revealed in several omics studies such as transcriptome analysis [54,55] and microbiome analysis [56]. In this study, we observed that 20.8% (33,107 genes) and 27.0% (4217) bovine genes were significantly DE between the pre-weaning (D33) and post-weaning (D96) periods in ileum and rumen tissues, respectively (Figure 1 and Figure 2, Supplementary Table S6). High variation of gene expression in rumen and ileum tissues between the pre- and post-weaning periods might reflect changes in anatomical size increases, metabolic changes as well as adaptation to different diets. Calves were fed with different diets (milk replacer and solid feed (starter diet and hay)) which require the activities/interactions of different groups of enzymes and pathways to digest and absorb them. As a consequence, distinct groups of genes and pathways are required in each period.

In rumen, several high DE genes are important for immune functions such as lymphocyte antigen 6 family member D (*LY6D*), mucin 1 (*MUC1*), embigin (*EMB*) and EYA transcriptional coactivator and phosphatase 2 (*EYA2*). LY6D protein initiates the first stage of B cell development [57] while MUC1 protein plays important roles in the first line defense against invading pathogens [58]. Meanwhile, *EMB* and *EYA2* have important roles in development processes [59,60]. As expected, DE genes were enriched in many biological processes related to regulation of biological and cellular processes, which reflect the major developmental changes in the physiology and functions of the rumen from pre- to post-weaning stages (Figure 1). Notably, top BP- and MF-GO terms are involved in G-protein-coupled receptors, which are important components of cell signaling and also vital processes required for the normal growth and development of cells [61]. Interestingly, top enriched MF-GO terms (e.g., poly(A) RNA binding, RNA binding, transcription factor binding and nucleic acid binding, etc.) for DE rumen genes involved in RNA processing reflects the many transcriptional activities that take place during growth and development. The most important pathway enriched for rumen DE genes was ribosomes, an important pathway for the regulation of protein biosynthesis. Other interesting pathways have roles in cellular signaling such as thyroid hormone, B cell receptor and Notch signaling pathways. The regulation of thyroid hormone is crucial for animals to respond to changes in nutrient availability and requirements and, to homeorhetic changes during different physiological stages [62]. Therefore, it is not surprising that this pathway was the most significantly enriched pathway in this study. Meanwhile, the enrichment of B cell receptor signaling pathway might reflect changes in immune activities in the rumen during the early period of growth, while the enrichment of Notch signaling pathways might reflect changes in cellular activities in rumen from the pre-weaning to the post-weaning stages.

In ileum, radical *S*-adenosyl methionine domain containing 2 (*RSAD2*) gene was the most significant DE gene between D33 and D96 (p.BH) = 6.05 × 10^−11^). This gene plays a key role in the innate immune response system and has a wide variety of antiviral activities [63]. Another most significant DE gene, 2′-5′-oligoadenylate synthetase 2 (*OAS2*) (p.BH = 2.34 × 10^−08^), functions as a regulator in the innate immune response process [64,65]. Similar to rumen DE genes, many ileum DE genes were enriched in BP-GO terms involved in cellular signaling and system development while MF-GO terms are involved in RNA processing and cell cycle regulation (Figure 2, Table S7c). Notably, several BP-GO terms (negative regulation of type I interferon production, interferon-beta production and positive regulation by symbiont of host I-κB kinase/nuclear factor-κB (NF-κB) cascade) and KEGG pathways (T cell receptor signaling pathway, B cell receptor signaling pathway and tumor necrosis factor (TNF) signaling pathway) related to immune activities were significantly enriched for ileum DE genes. Previously, Malmuthuge et al. [66] examined the expression of immune related genes (Toll-like receptors (TLR), peptidoglycan recognition protein 1 (*PGLYRP1*) and antimicrobial peptides (β-defensin)) in GIT tissues from pre- (three weeks of age) and post-weaned (six weeks of age) calves and reported a down regulation of most TLR genes after weaning, coinciding with a drastic change in the microbiome, as well as the morphological and functional changes in the GIT. These results may reflect a developmental shift toward that of a mature ruminant GIT to prevent unnecessary inflammatory responses to a shifting intestinal microbiome [66]. However, we did not observe significant differential expression of TLR genes between the pre- and post-weaning periods in this study. In fact, the most important pathway enriched for DE genes in ileum was ubiquitin mediated proteolysis. Protein ubiquitination mainly functions as a signal for 26S proteasome dependent protein degradation and therefore plays an important role in eukaryotic cellular processes. Another interesting enriched pathway was insulin signaling pathway which was not enriched for rumen DE genes. The insulin signaling pathway is crucial for carbohydrate metabolism. Therefore, the expression of genes related to this process was changed more in the Ileum than in the rumen. Using functional prediction from microbiome data, Meale et al. [67] reported that genes related to carbohydrate metabolism decreased post-weaning suggesting a shift in nutrient metabolism away from the lower GIT to ruminal fermentation. Some other significantly enriched pathways were related to growth control and disease (e.g., mechanistic target of rapamycin (mTOR) signaling pathway), regulation of cell cycle (e.g., cell cycle pathway) and cell matrix adhesions (e.g., focal adhesion pathway).

### 4.2. lncRNAs in Rumen and Ileum Tissues and Predicted Functions

In this study, we identified 1567 (62 known and 1505 novel) and 4243 (88 known and 4155 novel) lncRNAs in ileum and rumen tissues, respectively (Supplementary Table S4). The differences in the number of identified lncRNAs in the rumen and ileum tissues might reflect their different roles in each tissue since lncRNAs are known to be tissue specific [4,20,21]. Similar to lncRNA studies in other bovine tissues [21,29,30,31,33] (the majority of identified lncRNAs were 200–999 bp long (44.70%) and composed of one transcript (95.85%). Moreover, in the same trend with other studies [4,21,28], the majority of identified lncRNA transcripts are located in the intergenic regions.

It is well documented that lncRNAs may act in *cis* or *trans* to regulate the activities of genes [15,68,69,70,71,72]. Due to current lack of information, the functions of bovine lncRNAs can be inferred either by their physical position to protein coding genes such as their potential *cis* target genes (genes neighboring lncRNAs) or by their co-expression relationship with mRNAs (guilt by association methods) [73,74,75,76]) or determined experimentally. To infer the function of lncRNAs, we identified their *cis* target genes (genes located within 50 Kb flanking regions of lncRNAs), especially those having significant co-expression correlation (p.BH < 0.05) with lncRNAs. The combination of two filtering options (distance and strength of correlation) strengthened the confidence of potential *cis* target gene prediction for these lncRNAs but might lead to the loss of information since there is no clear definition of the distance from lncRNAs that should qualify a gene as its *cis* target. Moreover, it should be noted that, the co-expression (guilt by association) analysis might not completely reflect the causal relationship between lncRNAs and mRNAs.

Nevertheless, we identified 632 potential *cis* target genes for 884 lncRNAs (Supplementary Table S8a) in the rumen. A most interesting potential *cis* target gene was *WWOX*, predicted as potential *cis* target gene to 27 different lncRNAs. This gene encodes a member of the short-chain dehydrogenases/reductases (SDR) protein family and is known as a tumor suppressor [77]. Furthermore, *WWOX* has been shown to be regulated by lncRNA rP11-190D6.2 [78]. Since *WWOX* was not significantly DE between pre- and post-weaning (p.BH = 0.13) periods, we suspect that it performs similar functions in both periods. Another interesting gene was bone morphogenetic protein receptor type 1A (*BMPR1A*), predicted as *cis* target of 13 lncRNAs (rXLOC_042928, rXLOC_042929, rXLOC_042930, rXLOC_042932, rXLOC_042934, rXLOC_042935, rXLOC_042936, rXLOC_042938, rXLOC_042939, rXLOC_042940, rXLOC_042942, rXLOC_042945 and rXLOC_042946) is important in Juvenile Polyposis Syndrome (JPS) in humans [79]. JPS is characterized by predisposition to hamartomatous polyps in the GIT [80]. Given the important function of *BMPR1A* gene in the GIT health of humans, further studies are required to establish it roles in the bovine GIT. rXLOC_044298 and rXLOC_036836 potentially *cis* targeted 13 and 6 mRNAs, respectively (Supplementary Table S8a) but none of them was significantly DE between the two periods. Therefore, further functional studies to characterise their roles are needed. rXLOC_036836 (NONBTAG014380.2) is a known lncRNAs located on bovine chromosome 5 (from 104,262,200 to 104,262,521 bp) but no report on it function in the rumen exist. Enrichment results indicated that a high number of potential *cis* target genes are involved in posttranscriptional gene silencing by RNA or microRNA processing. Although the interaction between lncRNAs and miRNAs is well documented [81,82], no information on such interaction is available in the rumen. The enrichment of potential *cis* target genes of lncRNAs suggest potential roles of lncRNAs in the regulation of cell cycle, metabolic process, cellular metabolic processes and cell macromolecule catabolic process (Figure 3) which are essential processes for normal development. Moreover, rumen lncRNAs play potential roles in the regulation of pathways involving energy sources (oxidative phosphorylation pathway), cell signaling (p53 signalling or ErbB signaling) and immunity (T cell receptor signaling, Fc-γ-mediated phagocytosis and NF-κB signaling) (Figure 4). The importance of these pathways in the development and functions of the rumen have been reported by several authors [83,84,85]. Perhaps, the most interesting results for rumen lncRNAs is the fact that several DE lncRNAs also potentially *cis* targeted mRNAs that were DE between pre- and post-weaning periods. A notable rumen DE lncRNA is rXLOC_025037, which also potentially *cis* targeted four DE genes (*WDR97*, *MAF1*, *EXOSC4* and *GPAA1*) (Figure 7). Glycosylphosphatidylinositol anchor attachment 1 (*GPAA1*) gene serve an important function in linking proteins to the cell surface membrane while exosome component 4 (*EXOSC4*) is related to cyclin-dependent kinases (CDK)-mediated phosphorylation and removal of cell division cycle 6 (CDC6) and deadenylation-dependent mRNA decay [86], however it is not clear how these genes function in the rumen.

In ileum, 610 lncRNAs potentially *cis*-targeted 439 mRNAs (Supplementary Table S8b) and *BMPR1A* and ST3 β-galactoside α-2,3-sialyltransferase 1 (*ST3GAL1*) were amongst genes potentially *cis*-targeted by the highest number of lncRNAs. The function of *BMPR1A* has been described above, while *ST3GAL1* is an important gene in cancer [87] but little is known about its functions in the ileum. Microtubule cytoskeleton organization and endomembrane system organization were the most significantly enriched GO terms for potential *cis* target genes of ileum lncRNAs (Figure 5, Supplementary Table S9c). These GO terms are essential to many cellular processes. Immune related GO terms such as modulation by virus of host morphology, physiology, lymphatic endothelial cell differentiation and regulation of viral release from host cell were also significantly enriched (Figure 5, Supplementary Table S9c) suggesting that ileum lncRNAs might have roles in the regulation of immunity pathways. Moreover, we also observed that inflammatory mediator regulation of transient receptor potential (TRP) channels pathway was significantly enriched for ileum lncRNAs potential *cis* target genes (*ADCY7*, *MAP2K6*, *MAPK8* and *PLCG1*). The mammalian TRP superfamily comprises 28 TRP cation channels [88] involved in the regulation of gastrointestinal motility and absorption [89]. Similar to rumen, ileum lncRNAs might also regulate genes in oxidative phosphorylation pathway and lipid metabolism related pathways (glycosphingolipid biosynthesis and phosphatidylinositol signaling system). Interestingly, three DE lncRNAs also potentially *cis* regulated the G-patch domain-containing 2 (*GPATCH2*) gene with potential roles in spermatogenesis [90] and tumor growth during breast cancer [91]. However, the function of this gene in ileum is still unknown. Another notable gene is cysteine- and glycine-rich protein 1 (*CSRP1*), a member of the cysteine-rich protein (CSRP) family involved in regulatory processes important for development and cellular differentiation. Variants of *CSRP1* have been associated with growth and carcass traits in cattle [92].

## 5. Conclusions

To the best of our knowledge, this is the first study to characterize the lncRNA expression in calf’s ileum and rumen tissues. This study has shown the potential molecular mechanisms and pathways involved in the early development of calf GIT. The enrichment of DE mRNAs confirmed the major drivers for the switch from pre- to post-weaning involved in the regulation of growth, development and immune function. Relevant genes for these processes such as *LY6D*, *MUC1*, *EMB* and *EYA2* in rumen and *RSAD2* and *OAS2* in ileum tissues were identified. This study also characterized lncRNAs that may be involved in developmental processes of calves GIT and identified potential lncRNAs and the potential *cis* target genes that might be important for the regulation of the development of the GIT. Therefore, this study has set a baseline for the further exploration of the roles of lncRNAs in calf’s GIT.

## Figures and Tables

**Table 1:** Top significant differentially expressed genes between day 33 (pre-weaning) and day 96 (post-weaning) in rumen and ileum tissues.

Ensemble Name	Gene Name	Base Mean	Log2FC ^1^	*p*-Value	p.BH ^2^
**Rumen**					
*ENSBTAG00000044010*	*EMB*	45.41	−2.85	3.43 × 10^−33^	2.68 × 10^−29^
*ENSBTAG00000034498*	*LY6D*	45.30	−2.74	9.13 × 10^−28^	4.76 × 10^−24^
*ENSBTAG00000016145*	*ADAMTS19*	281.43	−1.30	9.85 × 10^−26^	3.85 × 10^−22^
*ENSBTAG00000013066*	*IGF2*	2769.08	−1.46	1.57 × 10^−23^	4.91 × 10^−20^
*ENSBTAG00000017104*	*MUC1*	18.85	−2.10	8.69 × 10^−21^	2.26 × 10^−17^
*ENSBTAG00000017032*	*FREM2*	344.45	−1.67	3.78 × 10^−19^	8.45 × 10^−16^
*ENSBTAG00000001382*	*SLC26A9*	26.28	−2.26	1.52 × 10^−18^	2.97 × 10^−15^
*ENSBTAG00000013336*	*EYA2*	5.79	−2.04	6.12 × 10^−18^	1.06 × 10^−14^
*ENSBTAG00000046549*	*BICD2*	785.41	0.42	5.94 × 10^−17^	9.28 × 10^−14^
*ENSBTAG00000048309*	*KIAA1024L*	12.77	−1.60	1.73 × 10^−16^	2.46 × 10^−13^
*ENSBTAG00000002847*	*D2HGDH*	305.50	0.98	2.26 × 10^−16^	2.94 × 10^−13^
*ENSBTAG00000037804*	*IKZF2*	73.24	−1.46	2.73 × 10^−16^	3.28 × 10^−13^
*ENSBTAG00000038093*	*PEG10*	91.51	−1.43	3.29 × 10^−16^	3.68 × 10^−13^
*ENSBTAG00000048213*	*ENSBTAG00000048213*	87.58	−0.68	3.71 × 10^−16^	3.86 × 10^−13^
*ENSBTAG00000015751*	*MEOX1*	47.50	−1.02	4.22 × 10^−16^	4.13 × 10^−13^
*ENSBTAG00000011171*	*PIEZO2*	194.57	−0.76	2.83 × 10^−15^	2.61 × 10^−12^
*ENSBTAG00000015303*	*MPP6*	35.74	−0.91	4.26 × 10^−15^	3.70 × 10^−12^
*ENSBTAG00000032821*	*SCEL*	95.16	−1.85	4.50 × 10^−15^	3.70 × 10^−12^
*ENSBTAG00000015402*	*ENSBTAG00000015402*	17.68	1.54	9.55 × 10^−15^	7.46 × 10^−12^
*ENSBTAG00000021420*	*EPHA7*	45.08	−1.43	1.76 × 10^−14^	1.31 × 10^−11^
*ENSBTAG00000018303*	*PAPPA2*	*52.76*	−*1.48*	1.95 × 10^−14^	1.39 × 10^−11^
**Ileum**					
*ENSBTAG00000016061*	*RSAD2*	562.08	−2.00	4.13 × 10^−15^	6.05 × 10^−11^
*ENSBTAG00000005057*	*FAM204A*	296.54	0.50	8.36 × 10^−13^	6.13 × 10^−09^
*ENSBTAG00000020142*	*LMBRD2*	296.05	0.53	2.39 × 10^−12^	1.17 × 10^−08^
*ENSBTAG00000011789*	*ENSBTAG00000011789*	410.62	0.50	1.22 × 10^−11^	2.34 × 10^−08^
*ENSBTAG00000012552*	*FMR1*	676.18	0.42	1.23 × 10^−11^	2.34 × 10^−08^
*ENSBTAG00000014628*	*OAS2*	243.63	−1.88	1.43 × 10^−11^	2.34 × 10^−08^
*ENSBTAG00000019059*	*ATG16L2*	319.11	−0.45	1.14 × 10^−11^	2.34 × 10^−08^
*ENSBTAG00000037527*	*OAS1X*	643.29	−1.54	1.38 × 10^−11^	2.34 × 10^−08^
*ENSBTAG00000038067*	*ENSBTAG00000038067*	124.15	−1.92	9.93 × 10^−12^	2.34 × 10^−08^
*ENSBTAG00000009677*	*PARP10*	704.04	−0.98	2.05 × 10^−11^	3.01 × 10^−08^
*ENSBTAG00000004934*	*NEMF*	958.94	0.46	8.49 × 10^−11^	1.13 × 10^−07^
*ENSBTAG00000042280*	*SNORA40*	55.73	0.85	1.46 × 10^−10^	1.65 × 10^−07^
*ENSBTAG00000043258*	*SNORA18*	33.13	1.05	1.44 × 10^−10^	1.65 × 10^−07^
*ENSBTAG00000014707*	*ISG15*	767.04	−1.75	1.95 × 10^−10^	1.96 × 10^−07^
*ENSBTAG00000019748*	*FAM208A*	2070.64	0.52	2.00 × 10^−10^	1.96 × 10^−07^
*ENSBTAG00000012907*	*ODF2L*	283.56	0.64	2.85 × 10^−10^	2.62 × 10^−07^
*ENSBTAG00000046797*	*NRAS*	1034.73	0.39	4.52 × 10^−10^	3.90 × 10^−07^
*ENSBTAG00000025029*	*MAN2A1*	1673.74	0.47	4.90 × 10^−10^	4.00 × 10^−07^
*ENSBTAG00000008048*	*GCFC2*	407.32	0.62	5.88 × 10^−10^	4.54 × 10^−07^
*ENSBTAG00000004593*	*TOP2B*	4025.86	0.35	7.58 × 10^−10^	5.56 × 10^−07^

^1^ Log2-fold change; ^2^ Corrected *p*-value using Benjamini and Hochberg correction.

**Table 2:** Top significantly differentially expressed lncRNAs between day 33 (pre-weaning) and day 96 (post-weaning) in rumen and ileum tissues.

LncRNA ^1^	Bta ^2^	Start	End	NONCODE Name ^3^	log2FC ^4^	FC ^5^	*p*-Value	p.BH ^6^
**Rumen**								
rXLOC_027852	17	59,152,438	59,152,902	Novel	−2.36	−5.13	9.59 × 10^−15^	4.07 × 10^−11^
rXLOC_020809	12	49,167,421	49,168,996	Novel	2.41	5.33	3.35 × 10^−14^	7.11 × 10^−11^
rXLOC_013111	9	70,095,274	70,095,655	Novel	−2.82	−7.06	1.61 × 10^−12^	2.27 × 10^−09^
rXLOC_025879	15	23,812,355	23,812,636	Novel	−0.83	−1.78	3.29 × 10^−12^	3.49 × 10^−09^
rXLOC_022247	13	18,885,057	18,885,409	Novel	2.61	6.11	9.49 × 10^−12^	8.05 × 10^−09^
rXLOC_035178	24	59,070,221	59,070,559	Novel	1.36	2.57	2.79 × 10^−11^	1.97 × 10^−08^
rXLOC_016360	11	80,849,366	80,849,978	Novel	1.79	3.45	1.02 × 10^−10^	6.12 × 10^−08^
rXLOC_041953	27	44,295,463	44,296,339	Novel	1.61	3.05	1.15 × 10^−10^	6.12 × 10^−08^
rXLOC_022213	13	18,879,759	18,880,268	Novel	2.16	4.47	2.45 × 10^−10^	1.05 × 10^−07^
rXLOC_033684	3	78,439,929	78,440,225	NONBTAG012391.2	1.05	2.07	2.47 × 10^−10^	1.05 × 10^−07^
rXLOC_039125	26	33,565,524	33,566,229	Novel	1.59	3.01	3.29 × 10^−10^	1.27 × 10^−07^
rXLOC_041968	27	44,323,639	44,323,953	Novel	1.39	2.63	3.86 × 10^−10^	1.36 × 10^−07^
rXLOC_006593	4	97,107,954	97,108,721	Novel	2.64	6.22	4.86 × 10^−10^	1.58 × 10^−07^
rXLOC_008000	7	95,774,878	95,775,405	Novel	1.26	2.40	1.68 × 10^−09^	4.95 × 10^−07^
rXLOC_022071	21	1,659,256	1,663,385	NONBTAG020155.1	2.03	4.09	1.85 × 10^−09^	4.95 × 10^−07^
rXLOC_035173	24	59,051,654	59,052,324	Novel	1.52	2.87	1.87 × 10^−09^	4.95 × 10^−07^
rXLOC_022254	13	18,885,611	18,886,350	Novel	2.49	5.61	2.47 × 10^−09^	6.17 × 10^−07^
rXLOC_022211	13	18,876,739	18,879,073	Novel	2.26	4.80	2.65 × 10^−09^	6.25 × 10^−07^
rXLOC_039103	26	33,517,784	33,518,636	Novel	1.37	2.59	2.90 × 10^−09^	6.47 × 10^−07^
rXLOC_045518	X	17,862,231	17,862,527	NONBTAG017530.2	1.73	3.31	3.18 × 10^−09^	6.74 × 10^−07^
**Ileum**								
iXLOC_002882	11	78,563,630	78,565,402	Novel	0.76	1.69	2.10 × 10^−08^	1.63 × 10^−05^
iXLOC_009320	16	21,758,311	21,761,092	Novel	0.68	1.60	2.65 × 10^−06^	1.03 × 10^−03^
iXLOC_007504	15	31,279,333	31,283,846	Novel	−1.15	−2.22	1.37 × 10^−05^	3.54 × 10^−03^
iXLOC_007519	15	32,192,174	32,195,976	Novel	0.66	1.58	9.51 × 10^−05^	1.48 × 10^−02^
iXLOC_033017	8	43,640,113	43,644,666	Novel	1.42	2.68	8.18 × 10^−05^	1.48 × 10^−02^
iXLOC_009321	16	21,761,255	21,764,378	Novel	0.60	1.51	1.82 × 10^−04^	2.36 × 10^−02^
iXLOC_008931	16	49,402,721	49,405,957	Novel	−0.88	−1.84	2.88 × 10^−04^	3.10 × 10^−02^
iXLOC_009324	16	21,772,956	21,777,346	Novel	0.48	1.40	3.41 × 10^−04^	3.10 × 10^−02^
iXLOC_031185	7	81,563,609	81,568,847	Novel	1.08	2.11	3.59 × 10^−04^	3.10 × 10^−02^
iXLOC_013444	19	63,355,768	63,357,128	Novel	−0.95	−1.93	4.56 × 10^−04^	3.13 × 10^−02^
iXLOC_013449	19	63,365,069	63,365,658	Novel	−1.04	−2.06	4.84 × 10^−04^	3.13 × 10^−02^
iXLOC_015752	20	63,795,770	63,798,826	Novel	−0.91	−1.88	4.69 × 10^−04^	3.13 × 10^−02^
iXLOC_013457	19	63,385,512	63,386,885	Novel	−1.07	−2.10	5.81 × 10^−04^	3.47 × 10^−02^
iXLOC_009284	16	21,647,689	21,649,116	Novel	0.76	1.69	7.07 × 10^−04^	3.93 × 10^−02^

^1^ Prefix r and i indicates lncRNAs identified in rumen and ileum tissues, respectively; ^2^ Bta: Bovine chromosome; ^3^ Novel indicates lncRNAs that have not been reported in NONCODE database; ^4^ Log2-fold change; ^5^ Fold change; ^6^ Corrected *p*-value using Benjamini and Hochberg correction.

**Table 3:** Differentially expressed lncRNAs and their corresponding differentially expressed potential *cis* target genes in ileum and rumen tissues.

LncRNA ^1^	Bta ^2^	Start	End	Ensembl Gene	Gene Symbol	Start	Stop	Cor ^3^	p.BH ^4^
**Ileum**									
iXLOC_007504	15	31,279,333	31,283,846	ENSBTAG00000021338	OAF	31,312,383	31,330,638	0.97	2.30 × 10^−10^
iXLOC_008931	16	49,402,721	49,405,957	ENSBTAG00000016057	CSRP1	49,332,770	49,353,517	0.83	7.72 × 10^−05^
iXLOC_009320	16	21,758,311	21,761,092	ENSBTAG00000001574	GPATCH2	21,783,189	21,808,519	0.84	3.86 × 10^−05^
iXLOC_009321	16	21,761,255	21,764,378	ENSBTAG00000001574	GPATCH2	21,783,189	21,808,519	0.75	9.20 × 10^−04^
iXLOC_009324	16	21,772,956	21,777,346	ENSBTAG00000001574	GPATCH2	21,783,189	21,808,519	0.92	6.39 × 10^−07^
**Rumen**									
rXLOC_001026	1	145,823,035	145,824,769	ENSBTAG00000021901	TRPM2	145,830,610	145,871,792	0.77	4.46 × 10^−07^
rXLOC_001033	1	146,129,434	146,206,714	ENSBTAG00000048090	UBE2G2	146,114,659	146,120,826	0.45	1.02 × 10^−02^
rXLOC_001744	1	88,589,659	88,590,085	ENSBTAG00000012463	ZMAT3	88,632,287	88,663,997	0.55	1.39 × 10^−03^
rXLOC_004066	11	88,446,930	88,450,243	ENSBTAG00000008160	MBOAT2	88,390,471	88,444,776	0.85	1.72 × 10^−09^
rXLOC_006791	7	13,559,789	13,561,916	ENSBTAG00000016352	STX10	13,548,241	13,551,708	0.78	2.51 × 10^−07^
rXLOC_006791	7	13,559,789	13,561,916	ENSBTAG00000018229	NFIX	13,596,367	13,658,112	0.58	5.62 × 10^−04^
rXLOC_006791	7	13,559,789	13,561,916	ENSBTAG00000019325	TRMT1	13,572,878	13,581,965	0.76	7.57 × 10^−07^
rXLOC_009274	8	63,568,396	63,573,554	ENSBTAG00000021367	ENSBTAG00000021367	63,617,041	63,630,082	0.74	2.49 × 10^−06^
rXLOC_010940	15	43,689,413	43,689,680	ENSBTAG00000016074	ENSBTAG00000016074	43,703,911	43,770,016	0.56	1.13 × 10^−03^
rXLOC_011271	9	71,006,423	71,217,097	ENSBTAG00000019460	MOXD1	71,245,109	71,341,930	0.57	7.51 × 10^−04^
rXLOC_016034	11	67,744,515	67,747,453	ENSBTAG00000017626	GFPT1	67,684,390	67,728,959	0.65	8.63 × 10^−05^
rXLOC_025037	14	1,907,631	1,909,665	ENSBTAG00000000658	WDR97	1,913,048	1,921,667	0.42	1.91 × 10^−02^
rXLOC_025037	14	1,907,631	1,909,665	ENSBTAG00000012242	MAF1	1,921,784	1,924,818	0.64	1.17 × 10^−04^
rXLOC_025037	14	1,907,631	1,909,665	ENSBTAG00000014607	EXOSC4	1,947,198	1,949,074	0.43	1.48 × 10^−02^
rXLOC_025037	14	1,907,631	1,909,665	ENSBTAG00000014610	GPAA1	1,942,672	1,945,910	0.42	1.77 × 10^−02^
rXLOC_025696	14	2,350,228	2,354,178	ENSBTAG00000014643	eef1d	2,317,971	2,326,718	0.44	1.42 × 10^−02^
rXLOC_029330	18	46,573,486	46,573,914	ENSBTAG00000002763	KMT2B	46,620,836	46,640,904	0.63	1.31 × 10^−04^
rXLOC_030259	19	25,389,242	25,389,587	ENSBTAG00000021091	ANKFY1	25,337,350	25,387,068	0.59	5.42 × 10^−04^
rXLOC_041027	27	25,434,455	25,440,584	ENSBTAG00000013581	LEPROTL1	25,422,502	25,432,327	0.53	2.25 × 10^−03^
rXLOC_041173	27	33,807,735	33,808,050	ENSBTAG00000003509	PLEKHA2	33,747,818	33,776,271	0.59	4.35 × 10^−04^
rXLOC_041174	27	33,813,850	33,814,207	ENSBTAG00000003509	PLEKHA2	33,747,818	33,776,271	0.65	8.60 × 10^−05^
rXLOC_042125	27	6,446,299	6,446,849	ENSBTAG00000004231	GPM6A	6,267,201	6,417,208	0.40	2.47 × 10^−02^
rXLOC_042503	27	32,987,255	32,989,352	ENSBTAG00000000978	ASH2L	32,989,769	33,014,982	0.74	1.74 × 10^−06^
rXLOC_045046	X	130,424,538	130,424,911	ENSBTAG00000003727	SH3KBP1	130,459,281	130,711,913	0.42	1.97 × 10^−02^
rXLOC_045053	X	130,432,550	130,433,452	ENSBTAG00000003727	SH3KBP1	130,459,281	130,711,913	0.53	2.12 × 10^−03^
rXLOC_045511	X	17,169,634	17,218,596	ENSBTAG00000020644	GPC4	17,087,447	17,122,426	0.73	2.75 × 10^−06^
rXLOC_045531	X	18,185,751	18,188,438	ENSBTAG00000030024	ENSBTAG00000030024	18,180,133	18,180,229	0.59	4.97 × 10^−04^

^1^ Prefix r and i indicates lncRNAs identified in rumen and ileum tissues, respectively; ^2^ Bta: Bovine chromosome; ^3^ The Person’s correlation coefficient; ^4^ Corrected *p*-value using Benjamini and Hochberg (BH) method.

## Supplementary Materials

The following are available online at http://www.mdpi.com/2073-4425/9/3/142/s1. Figure S1: KEGG pathways enriched for differentially expressed mRNAs in rumen tissue, Figure S2: KEGG pathways enriched for differentially expressed mRNAs in ileum tissue, Table S1: Genes and primer sequences used in qPCR validation of RNA-Sequencing data, Table S2: RNA-Seq read mapping statistics, Table S3: Genes (mRNA) in (a) rumen and (b) ileum tissues retained for further analyses, Table S4: Identified lncRNA genes in ileum and rumen tissues of calves. (a) Ileum known lncRNAs; (b) Ileum novel lncRNAs; (c) Rumen known lncRNAs; (d) Rumen novel lncRNAs; (e) LncRNAs common to ileum and rumen tissues, Table S5: Genomic features and characteristics of identified lncRNA. (a) Transcript length distribution of lncRNAs; (b) Chromosomal distribution of identified lncRNA genes and transcripts in ileum and rumen tissues of calves; (c) Number of transcripts per lncRNA gene and exons per lncRNA transcript; and (d) LncRNA classes based on genomic location, Table S6: Differentially expressed mRNAs in (a) ileum and (b) rumen tissues and (c) common DE mRNAs, Table S7: (a) GO terms enriched for rumen DE mRNAs; (b) KEGG pathways enriched for rumen DE mRNAs; (c) GO terms enriched for ileum DE mRNAs; (d) KEGG pathways enriched for ileum DE mRNAs, Table S8: Potential *cis* target genes of lncRNAs in (a) rumen and (b) ileum tissues and (c) differentially expressed lncRNAs and their differentially expressed mRNAs, Table S9: (a) GO terms enriched for rumen lncRNA potential *cis* target genes; (b) KEGG pathways enriched for rumen lncRNA potential *cis* target genes; (c) GO terms enriched for ileum lncRNA potential *cis* target genes; (d) KEGG pathways enriched for ileum lncRNA potential *cis* target genes, Table S10: Differentially expressed lncRNAs between day 33 and day 96 in rumen (a) and ileum (b) and potential *cis* target mRNAs for DE lncRNAs in (c) rumen and (d) ileum tissues.

## Availability of Sequence Data

The sequence data has been submitted to Gene Expression Omnibus database (BioProject PRJNA400067) and it is available through this link: https://www.ncbi.nlm.nih.gov/bioproject/400067.
