# Supplementary material for: Integration of lncRNA and mRNA Transcriptome Analyses Reveals Genes and Pathways Potentially Involved in Calf Intestinal Growth and Development during the Early Weeks of Life
Source: Genes (Basel). 2018 Mar 5;9(3):142. doi: 10.3390/genes9030142 (PMC5867863; doi:10.3390/genes9030142)
Supplement: Supplementary file 1 [file genes-09-00142-s001.zip › Table S1_Genes and qPCR primer sequences..docx]

**Table S1: Genes and primer sequences used in qPCR validation of RNA-sequencing data**

| Gene | Accession number | Primer | Primer Concentration (nM) | Primer sequence | Amplicon length |
| --- | --- | --- | --- | --- | --- |
| GPI | NM_001040471.1 | Forward | 300 | 5’-CTT CTG CAG TCG CGA AAC AC-3’ | 102 |
|  |  | Reverse | 300 | 5’-CCC AAT CCC AGA ACT CGA ACA-3’ |  |
| ATP5B | NM_175796.3 | Forward | 300 | 5’-ATC ACC ACC ACC AAA AAG GGA-3’ | 108 |
|  |  | Reverse | 300 | 5’-AGC ATC CAA ATG GGC AAA GG-3’ |  |
| PGK1 | NM_001034299.1 | Forward | 300 | 5’-ACA AGC TGA CTC TGG ACA AGC-3’ | 113 |
|  |  | Reverse | 300 | 5’-AGC CTT GAT CCT CTG GTT GTT-3’ |  |
| PPIB | NM_174152.2 | Forward | 300 | 5’-AGA CAG CAA ATT CCA TCG TGT G -3’ | 83 |
|  |  | Reverse | 300 | 5’-AGC GTT CAC CGT AGA TGC TC -3’ |  |
| OAS1X | NM_178108.2 | Forward | 300 | 5’-CGA CCA GGA GGT GGA GTT CG -3’ | 98 |
|  |  | Reverse | 300 | 5’-TGA GCT TCA CGT AGA TTT GAG GG -3’ |  |
| OAS2 | NM_001024557.1 | Forward | 300 | 5’-CTC AGG CAG AAC GAG TCA CC -3’ | 120 |
|  |  | Reverse | 900 | 5’-GAA AGT CTG GTT GGG CTG GA -3’ |  |
| IFI6 | NM_001075588.1 | Forward | 300 | 5’-TCT TTC TCT GCT CTC CTC CAA G -3’ | 113 |
|  |  | Reverse | 300 | 5’-GCA GCC GCA GGT GTA GAG TA -3’ |  |
| MX1 | NM_173940.2 | Forward | 300 | 5’-TCG AGG ATC ACG CAC ATT TC -3’ | 120 |
|  |  | Reverse | 300 | 5’-CCA ACA GGG GCA GAG TTT TA -3’ |  |
| UBA7 | NM_001012284.1 | Forward | 300 | 5’-GCT ATC TGC ACC TGG CTG AA -3’ | 89 |
|  |  | Reverse | 300 | 5’-GCA GGT CCA CTT CAG GTG AT -3’ |  |
| ACTA2 | NM_001034502.1 | Forward | 300 | 5’-AAG CCC AGC CGA GAA CTT T -3’ | 99 |
|  |  | Reverse | 300 | 5’-CCA GAG CCA TTG TCA CAC AC -3’ |  |
| CA3 | NM_001034437.1 | Forward | 300 | 5’-TGC AGG GTT GTG TTT GAT GAT ACT T -3’ | 120 |
|  |  | Reverse | 300 | 5’-CGA GCC GTG ATC GTC CGA G -3’ |  |
| HERC6 | NM_001192644.2 | Forward | 300 | 5’-GGC CCT CAG TGG GAA TAA CG -3’ | 80 |
|  |  | Reverse | 300 | 5’-AAA CCA CAC CGA GAG TCT TCA -3’ |  |
| rXLOC_042149 | BTA 8: position  69609679 to 69618605 | Forward | 300 | 5’-GTG TAC AAG CCA AGG AGA AC -3’ | 157 |
|  |  | Reverse | 900 | 5’-GAC TAG CTA TGG CAG AGA ACT A -3’ |  |
| rXLOC_022071 | BTA 21, position  1659256 to 1663385 | Forward | 300 | 5’-CTC CTG ATG AGC TTC AGG TTT A -3’ | 127 |
|  |  | Reverse | 300 | 5’-GGT CCA TGT AAC AAG TAT TAA CAG AG -3’ |  |
