# Supplementary material for: Integration of lncRNA and mRNA Transcriptome Analyses Reveals Genes and Pathways Potentially Involved in Calf Intestinal Growth and Development during the Early Weeks of Life
Source: Genes (Basel). 2018 Mar 5;9(3):142. doi: 10.3390/genes9030142 (PMC5867863; doi:10.3390/genes9030142)
Supplement: Supplementary file 1 [file genes-09-00142-s001.zip › Table S2_Mappin statistics.docx]

Table S2: RNA-Seq read mapping statistics

|  | Rumen | Ileum | Total/rate |
| --- | --- | --- | --- |
| Total reads | 1,383,732,850 | 1,130,200,180 | 2,513,933,030 |
| Reads after adaptor/quality trimming | 1,322,642,346 | 1,082,243,572 | 2,404,885,918 |
| Rate after adaptor/quality trimming | 95.787% | 96.213% | 96.000% |
| Mapped reads | 1,168,494,785 | 1,025,936,631 | 2,194,431,416 |
| Mapping rate | 88.842% | 94.843% | 91.843% |
| Uniquely mapped reads | 978,683,760 | 900,097,472 | 1,878,781,232 |
| Rate of unique mapped reads | 84.206% | 88.135% | 86.172% |
