# Supplementary figures and images for: Integration of lncRNA and mRNA Transcriptome Analyses Reveals Genes and Pathways Potentially Involved in Calf Intestinal Growth and Development during the Early Weeks of Life
Source: Genes (Basel). 2018 Mar 5;9(3):142. doi: 10.3390/genes9030142 (PMC5867863; doi:10.3390/genes9030142)

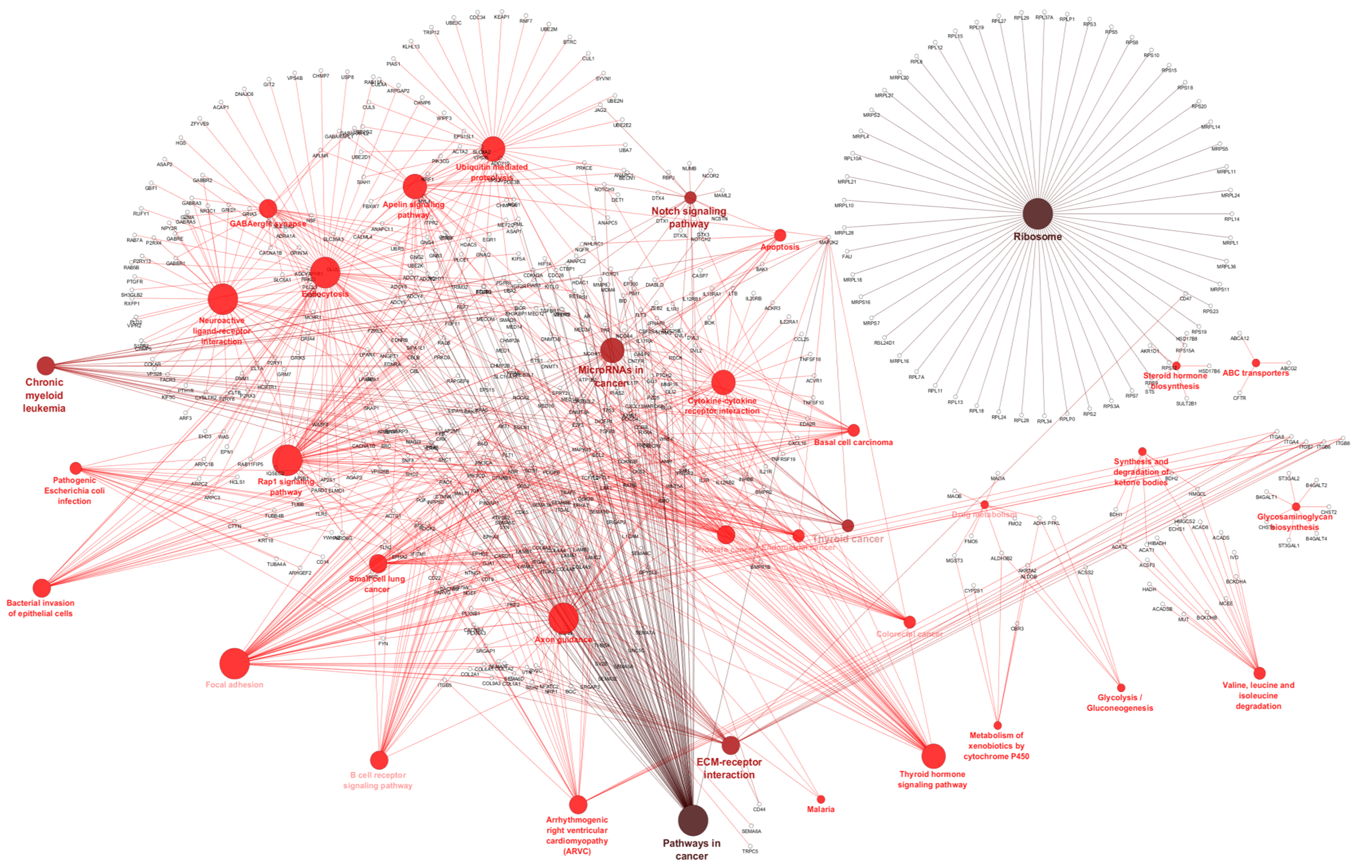

Supplement: Supplementary file 1 [file genes-09-00142-s001.zip › Figure S1. KEGGS pathways enriched for RumenDE mRNA.tif]

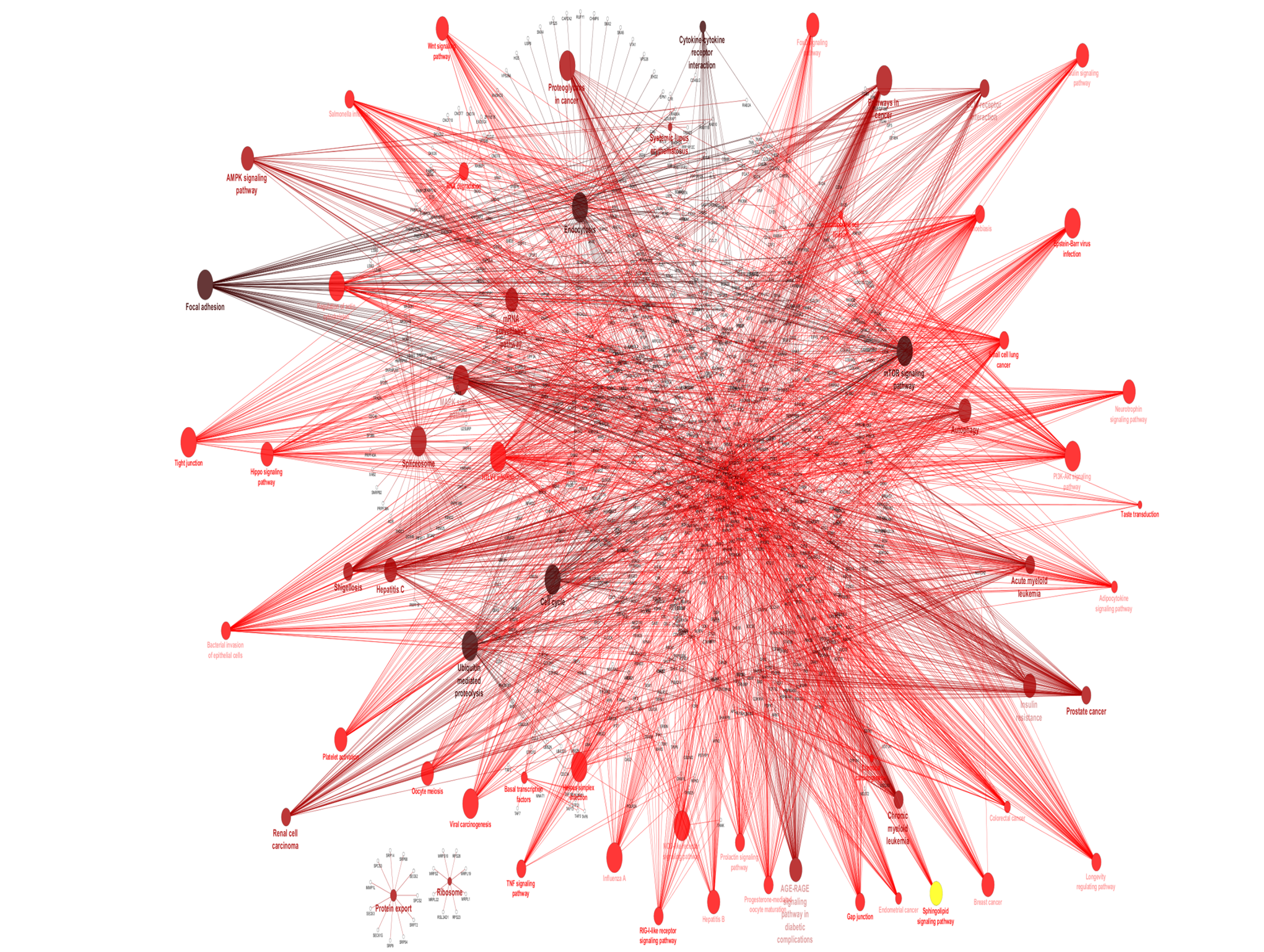

Supplement: Supplementary file 1 [file genes-09-00142-s001.zip › Figure S2. KEGGS pathways enriched for Ileum DE mRNA.tif]
